# Platelet-Rich Fibrin Facilitates Rabbit Meniscal Repair by Promoting Meniscocytes Proliferation, Migration, and Extracellular Matrix Synthesis

**DOI:** 10.3390/ijms18081722

**Published:** 2017-08-07

**Authors:** Chin-Chean Wong, Tzong-Fu Kuo, Tsung-Lin Yang, Yang-Hwei Tsuang, Ming-Fang Lin, Chung-Hsun Chang, Yun-Ho Lin, Wing P. Chan

**Affiliations:** 1 Graduate Institute of Clinical Medicine, College of Medicine, National Taiwan University, Taipei 10002, Taiwan; b8701153@tmu.edu.tw; 2 Department of Orthopedics, Shuang Ho Hospital, Taipei Medical University, New Taipei City 23561, Taiwan; tsuangyh@s.tmu.edu.tw; 3 Department of Orthopedics, School of Medicine, College of Medicine, Taipei Medical University, Taipei 11031, Taiwan; 4 Department of Post-Baccalaureate Veterinary Medicine, Asia University, 500, Lioufeng Road, Wufeng, Taichung 41354, Taiwan; 5 Research Center for Developmental Biology and Regenerative Medicine, National Taiwan University, Taipei 10617, Taiwan; yangtl@ntu.edu.tw; 6 Department of Otolaryngology, National Taiwan University Hospital and College of Medicine, Taipei 10051, Taiwan; 7 Department of Radiology, Wan-Fang Hospital, Taipei Medical University, 111 Hsing Long Road, Section 3, Taipei 11651, Taiwan; afun@w.tmu.edu.tw; 8 Department of Medical Imaging and Radiological Technology, Yuanpei University, Hsinchu 30015, Taiwan; 9 Department of Orthopedics, National Taiwan University Hospital, Taipei 10048, Taiwan; johnsonchchang@yahoo.com; 10 Department of Pathology, Taipei Medical University Hospital, Taipei Medical University, Taipei 11031, Taiwan; kevinyhl@tmu.edu.tw; 11 Department of Radiology, School of Medicine, College of Medicine, Taipei Medical University, Taipei 11031, Taiwan

**Keywords:** meniscocytes, meniscus, platelet-rich fibrin, regeneration

## Abstract

Although platelet-rich fibrin (PRF) has been used in clinical practice for some time, to date, few studies reveal its role as a bioactive scaffold in facilitating meniscal repair. Here, the positive anabolic effects of PRF on meniscocytes harvested from the primary culture of a rabbit meniscus were revealed. The rabbit meniscocytes were cultured with different concentrations of PRF-conditioned medium, and were evaluated for their ability to stimulate cell migration, proliferation, and extracellular matrix formation. In vivo, meniscal defects were created via an established rabbit animal model and were evaluated by a histology-based four-stage scoring system to validate the treatment outcome three months postoperatively. The in vitro results showed that PRF could induce cellular migration and promote proliferation and meniscocyte extracellular matrix (ECM) synthesis of cultured meniscocytes. In addition, PRF increased the formation and deposition of cartilaginous matrix produced by cultured meniscocytes. Morphological and histological evaluations demonstrated that PRF could facilitate rabbit meniscal repair. The data highlight the potential utility of using PRF in augmenting the healing of meniscal injuries. These advantages would benefit clinical translation, and are a potential new treatment strategy for meniscal repair.

## 1. Introduction

The knee meniscus acts as load bearer, shock absorber, joint lubricator, and stabilizer of the knee joint [1]. Many meniscal injuries to the knee are due to trauma resulting in instability and a loss of joint function. They can lead to pain or disability, degenerative joint changes, and symptomatic osteoarthritis [1,2]. The knee meniscus is not entirely vascularized, and blood is supplied only to the outer third of its structure. As a result, meniscal tissue has a limited potential for spontaneous repair [3].

Currently, most meniscal injuries are treated by a partial or complete meniscectomy or by suture repair when possible [4]. Previous studies have demonstrated a direct relationship between a partial or total meniscectomy and the development of osteoarthritis [4,5] Therefore, a ruptured meniscus is better treated by repair rather than removal, but the prerequisite for this treatment may rely on (1) a scaffold for cell adherence, (2) biological signals to stimulate cell migration and extracellular matrix synthesis, and (3) a sufficient number of cells to participate in tissue healing. Previous authors have suggested the usefulness of fibrin clots as scaffolds and stimuli for cell recruitment to appropriate sites of tissue regeneration [6,7,8]. Currently, platelet-rich plasma (PRP) combined with biodegradable hydrogel has been used to regenerate injured meniscus in vivo, and the results are promising [9]. Some clinical studies have further evaluated the role of PRP in augmenting adult meniscal healing, but the surgical outcomes are variable [9,10].

The major drawback of using PRP clinically is that when PRP is applied via liquid injection, the required mechanical environment of meniscal regeneration is difficult to achieve. Moreover, PRP implanted with either artificial or biological scaffolds may incur unexpected risk and lack integration. Unlike PRP, which needs additional scaffold for tissue in situ transplantation, platelet-rich fibrin (PRF) is a strictly autogenous fibrin-based biomaterial that encourages microvascularization and enables the local and progressive delivery of growth factors, which can be used to enhance bone and articular cartilage regeneration [11,12,13,14]. Scanning electron microscopy evaluations revealed that PRF has a honeycomb microstructure with platelets aggregated along the fibrin skeleton. The formation of a three-dimensional fibrin network resulted in the incorporation of cytokines into its mesh architecture [15]. PRF scaffolds enriched with growth factors are therefore effective in enhancing healing by sustainably providing anabolic cytokines to nourish cells. Moreover, PRF could also promote angiogenesis because it has a low thrombin level optimal for the migration of endothelial cells and fibroblasts, which is of paramount importance for meniscal healing [16].

Bioactive growth factors or cytokines are essential for tissue repair and regeneration. These essential factors release from the activated platelets after injury, inflammation, and tissue repair [17,18] Numerous studies have confirmed the effect of transforming growth factor β (TGF-β), basic fibroblast growth factor (bFGF), insulin-like growth factor I (IGF-1), vascular endothelial growth factor (VEGF), and platelet-derived growth factor (PDGF) in the repair and regeneration of cartilaginous tissues [19,20,21,22,23]. Thus, the purpose of this study was to investigate the effectiveness of PRF for the promotion of meniscal tissue healing. In our in vitro study, we first showed that PRF promoted meniscocytes migration, proliferation, and extracellular matrix synthesis. We also examined the regenerative effects of PRF in augmenting meniscal tissue healing with rabbit meniscal defects in vivo.

## 2. Results

### 2.1. In Vitro

#### 2.1.1. PRF Promoted In Vitro Wound Healing and Migration of Meniscocytes

**Figure 1:**
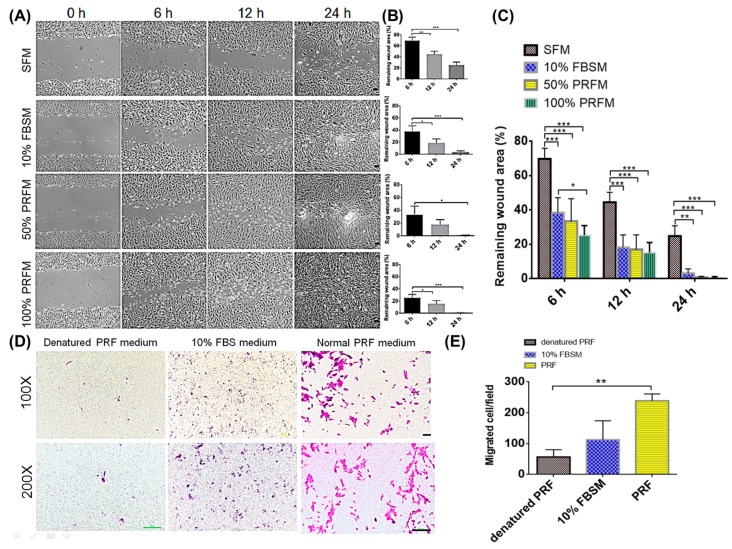
Platelet-rich fibrin (PRF) promotes monolayer wound healing of rabbit meniscocytes. (**A**) Phase micrographs of meniscocytes at 0, 6, 12, and 24 h after monolayer wounding. Scale bar: 100 μm; (**B**,**C**) Quantitation of cell migration using the monolayer wound healing assay. Data are the mean values ± standard deviation of four measurements for each time point and condition (*n* = 4); (**D**) Photomicrographs of the migration ability of each cell group are presented (upper panel: 100× magnification, lower panel: 200× magnification). Scale bar: 100 μm; (**E**) Migration assay data are representative of three experiments and are expressed as mean values ± standard deviation. * *p* < 0.05; ** *p* < 0.01; *** *p* < 0.001. SFM, serum-free medium; PRFM, PRF conditioned medium; FBSM, foetal bovine serum supplemented medium.

The effects of PRF on meniscocytes migration were first investigated using a scratch migration assay at three different time points (6, 12, and 24 h). Briefly, meniscocytes were isolated and cultivated with the indicated experimental conditions prepared from PRF. The cultivation prepared with serum-free medium (SFM) and 10% foetal bovine serum supplemented medium (FBSM) were used for comparison. Figure 1B shows the wound healing rates differs among each group from 6 h to 24 h. At 6, 12 and 24 h, meniscocytes treated with either 10% FBSM, 50% PRF conditioned medium (PRFM), or 100% PRFM had significantly better wound filling rates than cells treated with SFM (Figure 1A–C). This suggested that PRFM (50%, 100% PRFM) and 10% FBSM showed a better supporting effect on migratory meniscocytes than SFM. These data also demonstrated that PRF could enhance the monolayer wound healing of meniscocytes. A Boyden chamber transwell migration assay was used to further confirm the PRF’s effects on meniscocytes mobility. Figure 1D shows that normal PRF promoted the migratory ability of meniscocytes in transwell migration assay when compared with cells treated with 10% FBSM and denatured PRF. The quantitative results of cellular migration in each group are shown in Figure 1E.

#### 2.1.2. The Dose-Dependent Effect of PRF on Cell Phenotype and Proliferation of Meniscocytes

**Figure 2:**
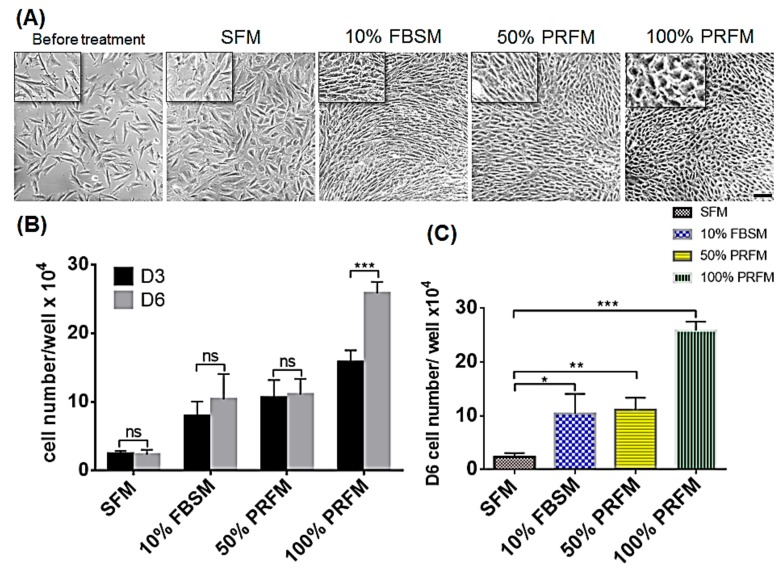
Effects of PRF medium on cell phenotype and proliferation of meniscocytes. (**A**) Representative phase-contrast images of cultured rabbit meniscocytes in serum-free medium (SFM), 10% foetal bovine serum (FBS) and proportionally diluted PRF medium (PRFM) on day 6. The meniscocytes in the SFM and 10% FBSM groups were extended and fibroblast-like cells, whereas the meniscocytes cultured with 100% PRFM were polygonal. Scale bar = 100 μm; (**B**) Cell proliferation analysis using the trypan blue exclusion method assessed the anabolic effects of SFM, 10% FBS, as well as 50% and 100% PRFM on cultured rabbit meniscocytes. The proliferative effects of FBS and different concentrations of PRF-conditioned media on rabbit meniscocytes were compared for 6 days; (**C**) On day 6, the cell number significantly increased after FBS and PRF stimulation. Data represent the measured cell numbers of various groups in triplicate assays (*n* = 3). (* *p* < 0.05; ** *p* < 0.01; *** *p* < 0.001). ns = not significant.

To confirm the effect of PRF on promoting cell anabolic activities, meniscocytes were cultured up to 6 days with either SFM, 10% FBSM, or PRFM in proportionally diluted concentrations (50% and 100%). The morphology, cell proliferation rate, accumulated glycosaminoglycan (GAG) levels, and chondrogenic protein deposition values were evaluated on days 3 and 6. Significant morphological alteration was noted in meniscocytes cultured under different conditions (Figure 2A). In terms of cell shape and appearance, most of the meniscocytes in the SFM group are rag-like in shape. Meniscocytes cultured with standard 10% FBSM were fibroblast-like cells. In contrast, meniscocytes cultured in 50% PRFM and 100% PRFM are more polygonal relative to cells cultivated in SFM and 10% FBSM (Figure 2A). Cell proliferation was evident after stimulation of rabbit meniscocytes with 10% FBSM as well as 50% and 100% PRFM (Figure 2B). On day 6, the cell number significantly increased in groups of 10% FBSM, 50% PRFM, and 100% PRFM when compared to the control. Of the PRFM groups, 100% PRFM increased the cell number the most over time compared to the SFM group (*p* < 0.001). The data confirmed that PRFM could significantly increase the number of meniscocytes with culture in a dose-dependent manner after 6 days culture (Figure 2C).

#### 2.1.3. PRF Anabolic Effects on Extracellular Matrix Synthesis of Meniscocytes

**Figure 3:**
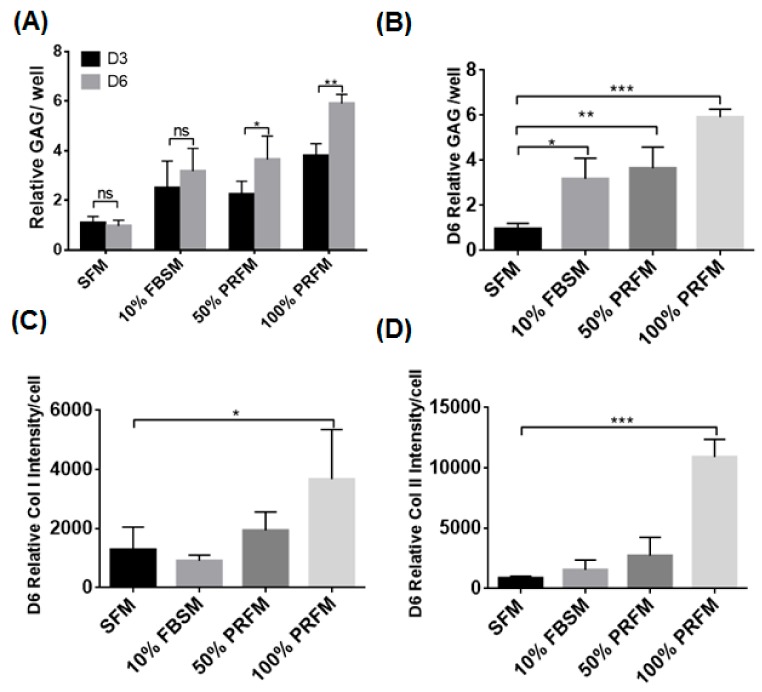
Effects of PRF on meniscocyte extracellular matrix (ECM) synthesis of cultured meniscocytes. (**A**) Synthesized GAG from cultured meniscocytes of each group on days 3 and day 6. The accumulated glycosaminoglycan (GAG) content was significantly higher in cells treated with 10% FBS as well as 50% and 100% PRF-conditioned media; (**B**) These cells exhibited significantly higher levels of accumulated GAG than the control (SFM group); (**C**,**D**) Meniscocytes treated with 100% PRFM for 6 days exhibited significantly higher levels of type I collagen (Col I) and type II collagen (Col II) deposition than the control. Data represent the quantified mean levels of Col I and Col II staining of various groups in triplicate assays (*n* = 3). (* *p* < 0.05; ** *p* < 0.01; *** *p* < 0.001). ns = not significant.

During the 6-day culture period, meniscocytes cultured in SFM and 10% FBSM expressed a low level of accumulated glycosaminoglycan (GAG) compared to cells cultured in 100% PRFM (Figure 3A). The GAG levels increased significantly from day 3 to 6 in cells cultivated in 50% and 100% PRFM. The results demonstrated the stimulatory effects of PRFM on GAG deposition in contrast to SFM and FBSM. At day 6, there was nearly a six fold higher anabolic capacity seen in meniscocytes cultured in 100% PRFM (5.91 ± 0.37) relative to those cultured in SFM (0.972 ± 0.23) (Figure 3B).

To further characterize the stimulatory effects of PRF on meniscocyte cartilaginous matrix formation, characteristic meniscal cell markers such as type I collagen (Col I) and type II collagen (Col II) depositions were measured in monolayer-cultured cells after 6 days culture. Figure 3C,D shows the intensity of immunohistochemical staining for Col I and Col II in different treatment groups after 6 days culture. The results showed that meniscocytes treated with 100% PRFM have a significantly elevated level of Col I and Col II deposition compared with SFM. At day 6, the measured intensity of Col I deposited by cells in SFM was 1291 ± 757 compared to the intensity of Col I deposited by 100% PRFM, which was 3667 ± 1685: a 2.8-fold difference in Col I expression (Figure 3C). On the other hand, the measured intensity of Col II deposited by cells in SFM was 867 ± 150, compared to cells in 100% PRFM which was 10901 ± 1451, a 12-fold difference in Col II expression (Figure 3D).

### 2.2. In Vivo

#### In Vivo Meniscal Repair Augmented by PRF

**Figure 4:**
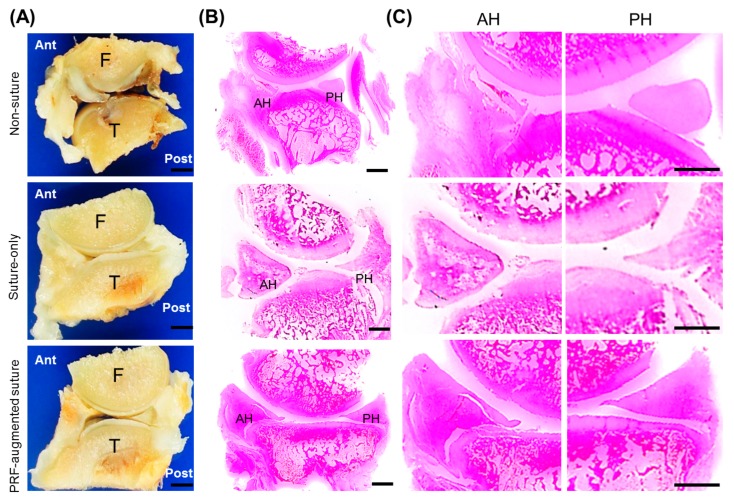
(**A**) Gross appearance of the representative rabbit knee joints from three groups (non-suture group, suture-only group, and PRF-augmented suture group) 3 months after operation; Scale bar = 10 mm (**B**) Macroscopic images of the representative rabbit knee joints from the non-suture group, suture-only group, and PRF-augmented suture group; Scale bar = 10 mm (**C**) Representative histological sections of meniscal defects of the anterior horn (AH) and posterior horn (PH) of medial meniscus from the non-suture group, suture-only group, and PRF-augmented suture group. Hematoxylin-eosin staining at low power magnification. Scale bar = 2.5 mm. F = Femur, T = Tibia.

**Figure 5:**
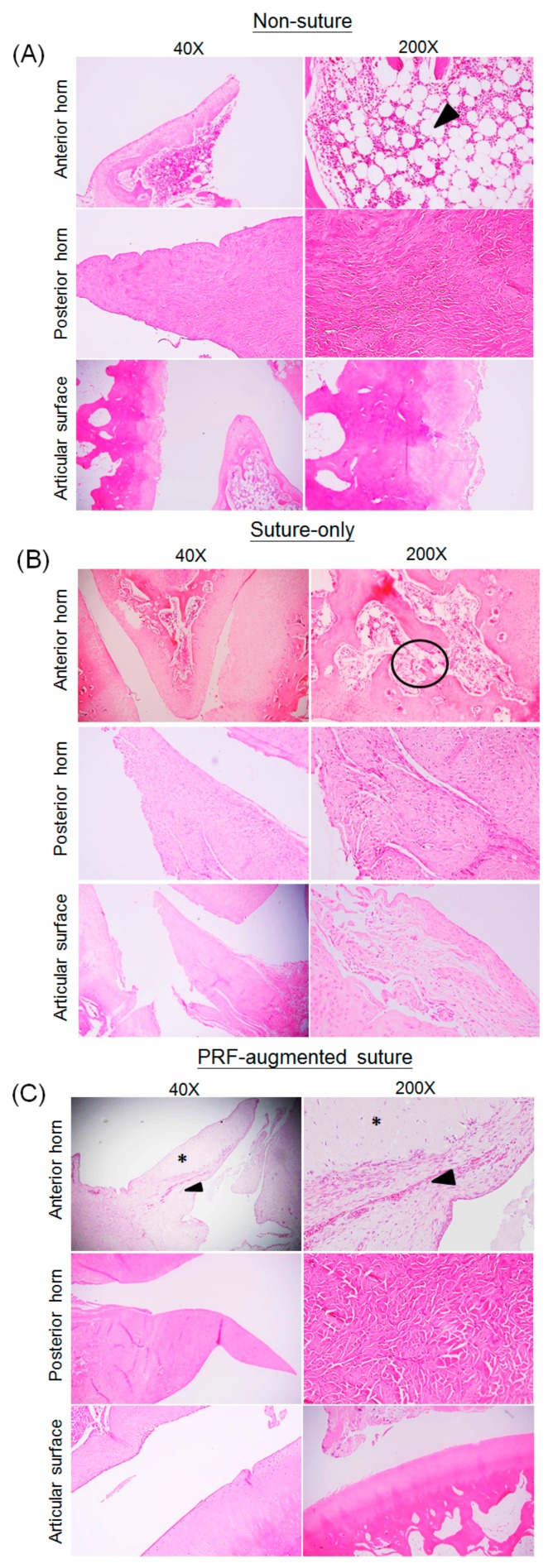

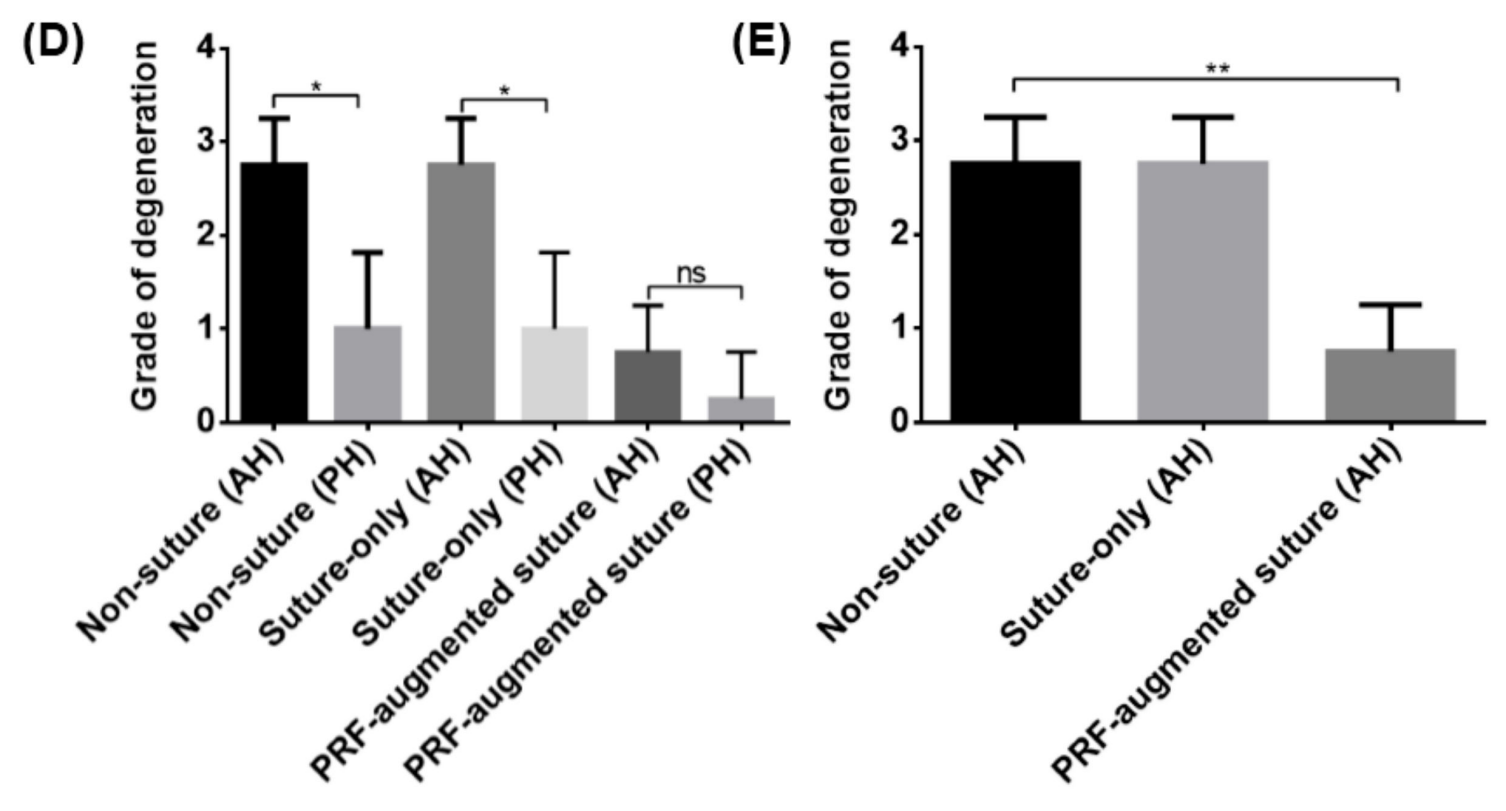
Histological evaluation of the degeneration in the medial menisci in the non-suture group, suture only group, and PRF-augmented suture group using a four-stage scoring system based on haematoxylin-eosin staining. (**A**) In the non-suture group (*n* = 6), separation of fibrocartilage with fat accumulation inside the meniscus (arrowhead) was observed; (**B**) In suture-only group (*n* = 6), degenerative changes such as matrix cleft with basophilic staining (circle) was observed; (**C**) In the PRF-augmented suture group (*n* = 6), most of the matrix was homogenously stained. Asterisk indicates the native meniscal tissue, arrowhead indicates the regenerated portion of meniscus (**D**) The grade of degeneration in AH and PH of all medial menisci were determined; (**E**) Significant degeneration was noted in the medial menisci in non-suture and suture-only groups. (* *p* < 0.05; ** *p* < 0.01). ns: not significant. AH: anterior horn; PH = posterior horn.

In the animal disease model, the meniscal defects were sutured and hand-diced PRF fragments were placed in or around the defect to enhance the healing response. The untreated group and suture-only group were used as controls for comparison. In each group, representative sections of medial meniscus (MM), anterior horn (AH), posterior horn (PH), and rabbit knee joint articular surface were demonstrated for comparison. Macroscopically, the AH of medial meniscus in the PRF-augmented suture group has better morphological integrity than the AH in the non-suture and suture-only groups, indicating that the meniscal defect has achieved better healing via PRF-augmentation (Figure 4A,B). Although the AH in the PRF-augmented suture group has significantly better defect healing in terms of gross morphology and histological scores, it was noted that specific regions of injured AH in the PRF-augmented suture group had not regenerated completely (Figure 4C). At higher magnification, it was noted that no signs of high-grade degeneration could be detected in either the AH or PH of medial meniscus in the PRF-augmented suture group. On the contrary, the MM in the non-suture and suture-only groups showed pronounced mucoid changes with clear signs of degeneration, such as matrix clefts and marked fat accumulations inside either the AH or PH (Figure 5A–C).

In summary, the AH in the PRF-augmented suture group showed a significantly lower degree of degeneration than the other two groups (Figure 5D,E). It was known that meniscal lesion could impair joint load distribution, and therefore initiates erosion of the adjacent articular surface. The histological results showed that articular cartilage in the PRF-augmented suture group has better congruity than joint space in the non-suture and suture-only groups, highlighting the importance of maintaining meniscal integrity. These data confirm the role of PRF in augmenting in vivo meniscal repair by promoting tissue healing and regeneration, and thereby preventing articular cartilage from overloading.

## 3. Discussion

Meniscal injuries are a common and important source of knee dysfunction. Nevertheless, effective treatment to promote meniscal healing is still lacking. As a consequence, arthroscopic resection of the torn meniscus remains the most common treatment, but the procedure would result in higher contact stresses at the articular surface of the knee, leading to the onset or progression of osteoarthritis [9,24,25,26]. Recently, meniscal repair instead of menisectomy has been advocated as an alternative, but has often failed for varied reasons. A lack of vasculature that provides intrinsic nutrition is believed to be one of the most important reasons for poor healing [3]. Some authors think that biologic factors might be of greater importance to the success of meniscal repair than the choice of the surgical technique [27,28]. The use of PRP has been demonstrated to enhance healing by introducing a higher concentration of growth factors to the injured region [9,10,29,30]. Both in vitro and in vivo studies have addressed the beneficial effects of PRP or PRP clots on meniscal repair [6,8,9,10,29,31,32,33], but to our knowledge, very limited studies concerning the role of PRF as a bioactive biomimetic scaffold in facilitating meniscal healing are available to date.

Unlike PRP, PRF slowly polymerizes during centrifugation and forms a gel-like tissue that is easily manipulated, and no additional additive for platelet activation is needed [11,12,13,14,34]. The established fibrin network supports cytokine enmeshment, cellular migration, and regional cell trapping. In the current study, we have demonstrated the positive stimulatory effects of PRF in promoting meniscocytes migration, proliferation, and matrix formation. Moreover, the preliminary in vivo results reveal the capacity of PRF to augment the healing of rabbit meniscal defects. We thus proposed to implant PRF at the site of damage during meniscal repair to provide a niche for meniscal cell migration, which would lead to better tissue healing.

The PRF scaffold acts as a reservoir of growth factors containing many kinds of anabolic cytokines, such as platelet-derived growth factor (PDGF), transforming growth factor-β (TGF-β), vascular endothelial growth factor (VEGF), and insulin-like growth factor-I (IGF-I) [19,20,21,22,23]. The release of growth factors from platelets has been associated with the initiation of a healing cascade leading to cellular chemotaxis, angiogenesis, collagen matrix synthesis, and cell proliferation. These bioactive molecules facilitate enhanced cell growth and proliferation, increased cartilaginous matrix production, and the upregulation of chondrogenic gene expression in PRF scaffolds [19,20,21,22,23]. Comparing to PRP, which releases a high concentration of growth factors in a relatively short period of time (minutes to hours), PRF displayed a continual and steady release of growth factors over a days to weeks period [35]. We believe that PRF sustainable growth factors that release kinetics would be more beneficial for meniscal healing, which usually takes days to weeks to complete.

Meniscal tissue healing is always limited by a paucity of vascularity, and it is thus important to have a sufficient blood supply in cell-based therapy [27,36]. Our results confirmed the efficacy of PRF in providing nutrient support to increase the number of cultured meniscocytes. The proliferative effects of PRF on rabbit meniscocytes in this current study concurs with other PRP-related in vitro studies using human meniscocytes, porcine meniscocytes, or human chondrocytes. On the other hand, from a morphological perspective, our results also revealed the effectiveness of PRF in maintaining the differentiation status of meniscocytes during monolayer culture without dedifferentiating into fibroblast-like cells, which would benefit meniscal tissue healing (Figure 2A).

Besides the impact on cell proliferation, it is particularly important to confirm that the PRF-based therapeutic approach to augment meniscal repair is facilitated by meniscocytes recruitment. The effects of PRP on cell recruitment and migration have been shown [37], but to further clarify whether similar effects happen in PRF, we performed an in vitro scratch wound healing assay and transwell migration assay to confirm the impact of PRF on the migratory activities of meniscocytes. In the scratch wound healing assay, PRFM at different concentrations (50% and 100%) could induce significant meniscocyte migration in culture similar to FBSM. In the transwell migration assay, normal PRF could successfully induce significant meniscocytes migration in culture, as that of PRP reported previously [38]. The growth factors and chemokines originating from PRF enhance the migration of meniscocytes [38]. Our results are comparable to other studies that showed the effects of fetal bovine serum on cell migration and the proliferation of porcine meniscal cells in microwound and repair models. On the other hand, meniscocyte extracellular matrix (ECM) formation is important for establishing the microenvironment for meniscal tissue repair and regeneration. Our results showed that PRFM stimulates the meniscocytes to synthesize significantly more glycosaminoglycan, collagen I, and collagen II compared to SFM, suggesting that PRF could serve as a bioactive scaffold by inducing regional meniscocytes chemotaxis, proliferation, and matrix synthesis. This would benefit a PRF-based therapeutic approach for augmenting in vivo meniscal repair.

Many biological approaches to improving the healing of meniscal defects have been reported, but with variable outcomes. Ishida et al. demonstrated positive results in meniscal punch defects by the application of PRP [29]. Toratani et al. reported that three-dimensional scaffold-free allogenic stem cells derived from adipose tissue successfully promoted meniscal healing in a rabbit model [39]. Hatsushika et al. reported that the intra-articular injection of synovial mesenchymal stem cells could promote meniscus regeneration, and protected articular cartilage in a massive meniscus defect model [40,41]. In contrast, Shin et al. showed that a single injection of leukocyte-rich PRP failed to enhance the healing of a horizontal tear of rabbit medial meniscus [42]. Zellner et al. also demonstrated the combination of PRP and composite matrices was not superior to its counterpart using bone marrow stem cells [31]. In summary, the outcomes of all of these studies have delineated the importance of cell sources that play a pivotal role in directing tissue healing. These findings lend further support to the fact that optimal meniscus healing could only be achieved in the presence of an appropriate cell type and a sufficient cell number to initiate tissue healing and neo-matrix synthesis. In the present study, the in vitro and in vivo data confirmed the concept that PRF could serve as a functional bioactive biomaterial that not only recruits regional cells but also creates a favorable microenvironment to benefit meniscocytes’ proliferation and differentiation. This concept concurs with previous reports revealing that a functional scaffold could facilitate the recruitment and homing of stem cells from adjacent tissues, and provide guidance for cellular differentiation [43,44]. It is noteworthy that a major obstacle hindering the effective translation of platelet-derivative biological therapy to augment meniscal healing is the inability to maintain the sustained level of cytokines necessary for tissue healing. Therefore, the combination of chemotactic and bioactive scaffolding advantages empowers PRF to be promising in developing a single-stage approach for meniscal repair.

It is known that a meniscal injury or defect leads to osteoarthritic changes in the joint [24,25,26]. A partial menisectomy causes a destabilization of the joint, leading to rapid degeneration and a more severe case of osteoarthritis than anterior cruciate ligament transection [45]. In the present study, particular attention was paid to evaluate the protective effect of PRF implantation for rabbit knee joints after meniscal defects were created. In Figure 5A,B (lower panel), it was shown that the articular surface was uneven with cartilage fibrillation or even detachment. There was a gap extended from the cartilage surface to the subchondral bone. On the contrary, in Figure 5C, the articular surface was relatively smooth with a limited degree of cartilage erosion. We considered that the superior healing process and chondroprotective phenomenon that occurred in the PRF-augmented suture group were attributable not only to the anabolic cytokines but also the presence of a number of anti-inflammatory mediators that can potentially affect numerous overlapping pathways simultaneously [46]. For instance, PRP was found effective in attenuating the effects of fibronectin fragments on both chondrocyte and meniscocytes production of procatabolic chemokines and matrix metalloproteinases [47]. In the future, the role of PRF in modulating inflammatory mediators warrants further in vitro and in vivo studies using large animals to further confirm the potential of clinical translation while exploring the underlying mechanisms.

However, there are some limitations to this study. First, the role of oxidative stress in affecting the inflammatory conditions in meniscal injuries was an important issue as previously reported [48]. Nonetheless, the role of PRF in modulating the inflammatory mediators was not fully investigated in this present study. Second, only six rabbits were used in each group, and only one time point was chosen for sacrifice: this resulted in somewhat low statistical power. Moreover, only one side of a rabbit’s knees was used in this study, which may raise concern about a lack of randomization, but was a consequence of the economic and ethical issues of animal studies. Third, we did not include imaging, such as magnetic resonance imaging (MRI), for pre-operative and post-operative outcome evaluation. Fourth, although the mechanical properties of the repaired meniscal tissues were not routinely measured as in previous studies, it is clinically important to determine the exact quality of the repaired tissues.

In conclusion, this study showed that PRF can promote meniscocytes migration, proliferation, and matrix formation. From this standpoint, PRF acts as a functional biomaterial that recruits cells and provides contact guidance for cellular differentiation. Compared to the non-suture or suture-only groups, the quality of meniscal healing significantly improved in the PRF-augmented suture group, possibly due to the integration of regional cells’ recruitment, proliferation, and differentiation. Based on the results, further studies will continue to examine long-term tissue healing in a larger animal model and perform preclinical evaluations necessary to fully validate this PRF-based therapeutic approach in a hope to expedite the clinical translation of meniscal repair.

## 4. Experimental Section

### 4.1. Study Design and Ethics Statement

All procedures of meniscocytes isolation and surgery on experimental animals were carried out according to the guide for the Care and Use of Laboratory Animals and approved by the Institutional Animal Care and Use Committee of National Taiwan University (IACUC approval number 99-092).

### 4.2. In Vitro

#### 4.2.1. Preparation of PRF-Conditioned Medium

**Figure 6:**
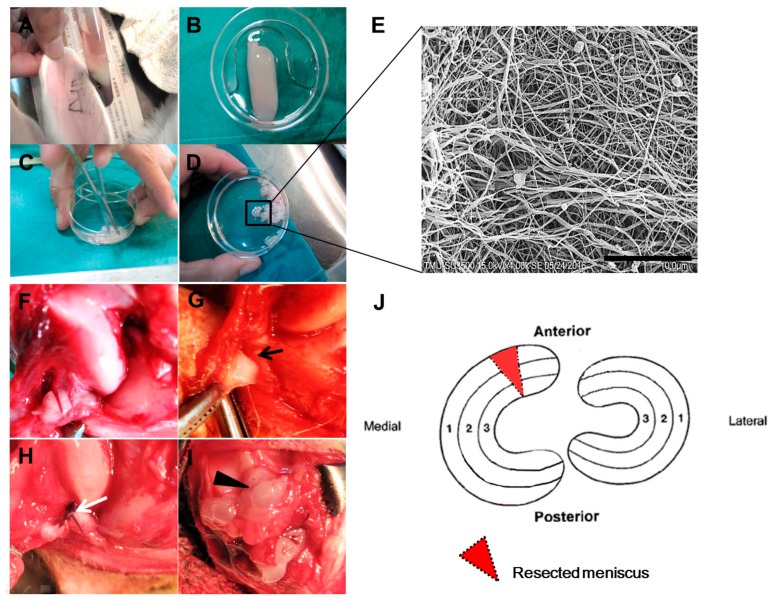
Photographs of PRF preparation and animal surgery. (**A**) The venous blood was centrifuged for separation to yield a PRF clot in the middle of the tube between the red blood cells and the platelet-poor plasma; (**B**) A stable PRF clot was separated from the red blood cells using a sterile syringe and scissors; (**C**,**D**) The PRF clots were minced into small pieces for implantation; (**E**) Scanning electron microscope picture showing the microstructure of PRF with honeycomb porous structure, Scale bar = 10 μm; (**F**,**G**) A 2.0 mm wedge shape defect was created at the anterior horn of the medial meniscus and a small portion of the meniscus was removed, black arrow indicating the meniscal defect; (**H**) The meniscal defect was approximated together in the suture-only group, with the white arrow indicating 5–0 prolene suture; (**I**) The meniscal defect was sutured and augmented with PRF, black arrowhead indicating diced PRF fragments; (**J**) Schematic illustration of wedge-shaped meniscal defect creation.

The PRF scaffold was prepared using the technique described by Choukroun et al. [15], venous blood was harvested from rabbits, stored in sterile tubes without anticoagulant supplement, and then centrifuged at 400× g for 10 min in a DSC-200A-2 table top centrifuge (Digisystem, Laboratory Instruments Inc., Taipei, Taiwan). The PRF scaffold was generated after these procedures, and was located between the red blood cells and the acellular plasma (Figure 6A,B). PRF samples were then stored at −80 °C until use. To prepare PRF-conditioned medium, the thawed PRF scaffolds were soaked in 10 mL of serum-free Dulbecco’s Modified Eagle’s Medium without foetal bovine serum supplement (DMEM/F-12 medium) (Gibco, Paisley, UK) in a centrifuge tube (defined as 100% PRF-conditioned medium). The tubes were then put into a tube rotator at 4 °C for 24 h. The conditioned medium was then collected and diluted to 50%. For in vivo experiment, the PRF samples were cut into small fragments with surgical scissors for later implantation in a PRF-augmented suture group (Figure 6C,D).

#### 4.2.2. Isolation and Culture of Rabbit Meniscocytes

Autologous rabbit menisci were harvested from the knee joints of 3 kg male New Zealand white (NZW) rabbits. The medial and lateral menisci were harvested from rabbit knee joints. The harvested menisci were placed in chilled Hank’s solution containing penicillin, streptomycin, and fungizone (P/S/F). The meniscal tissue was then diced into small fragments and digested with 0.05% (*w*/*v*) collagenase II (Gibco) for 18 h at 37 °C. The digestion suspension was washed via centrifugation (5 min at 1200 rpm) in DMEM/F-12 medium containing 10% foetal bovine serum (FBS) (Gibco). The cells were then plated at a density of 5 × 10^5^ cells/10 cm standard tissue culture plate (Orange Scientific, Braine-l’Alleud, Belgium) in 10 mL of culture medium and cultured in a 5% CO_2_ and 90% humidity incubator at 37 °C for several days until confluence was reached. The medium was changed every 3 to 4 days. When the cells reached 80% confluence, they were trypsinized and transferred into new 10 cm dishes with a density of 5 × 10^5^ cells/dish. The cells were sub-cultured until passage 1 (P1). The cell morphology was observed throughout the entire culture interval using inverted light microscope (Olympus, Tokyo, Japan). Morphological changes, such as cell shapes, sizes, and cell number were recorded at both 40× and 200× magnifications on the first, third, and sixth days of each passage.

#### 4.2.3. PRF Effects on Meniscocytes Migration and Wound Healing

P1 meniscocytes were seeded into 24-well tissue culture plate and cultured in DMEM medium with 10% serum supplement until reaching 80% confluence. Then, a 1 mL pipette tip was used to gently and slowly scratch the monolayer across the centre of the well. The wells were then washed twice with phosphate-buffered saline (PBS) after scratching to remove the detached cells. Then, each well was replenished with either serum-free medium (SFM), medium supplemented with 10% FBS (10% FBSM), 50% PRF conditioned medium (50% PRFM), or 100% PRF conditioned medium (100% PRFM). The cells were then observed for their migration at several time points (6, 12, and 24 h). The gap distance was quantitatively evaluated using Image J analytic software. To further confirm the effects of PRF in directing meniscocytes migration, a Bodyden chamber transwell migration assay was performed according to the manufacturer’s recommendation. Briefly, the 8 μm pore size cell culture inserts with polyethylene terephthalate membrane (BD Falcon, BD biosciences, Heidelberg, Germany) were used to analyse the migration of meniscocytes. P1 meniscocytes cultured in DMEM medium with 10% serum supplement were trypsinized and resuspended in serum-free medium. Then, the meniscocytes were seeded into inserts at concentration of 2 × 10^4^ cells/insert in 24-well chambers. Later, the outer chamber was filled in with either medium with 10% FBS (10% FBSM); serum-free medium containing PRF, or serum-free medium containing heat-denatured PRF. The plates were placed in the incubator at 37 °C in 5% CO_2_ and culture for 24 h. The non-migrated cells were removed from the upper surface of the membrane with a cotton swab. After rinsing twice with PBS and fixed with 4% paraformaldehyde, the inserts were stained with crystal violet. The numbers of migrated cells were counted using Image J analytic software.

#### 4.2.4. Proliferation of Meniscocytes

The P1 meniscocytes were suspended in DMEM/F12 medium at a density of 1 × 10^4^ cells/mL per well and loaded on 24-well plate. After 24 h, the medium was removed and 1 mL of SFM, 10% FBSM, 100% PRFM, or 50% PRFM were added, respectively. The medium was changed every 3 days, for a total culture period of 6 days. Cell morphology alterations were recorded throughout the entire culture interval using an inverted light microscope and digital camera. To further evaluate the effects of PRF on cell proliferation, the viable cell number in the respective groups were counted using trypan blue exclusion method in quadruplicate at each time point (day 3 and 6).

#### 4.2.5. Glycosaminoglycan (GAG) Synthesis of Cultured Meniscocytes

The accumulated GAG level was measured via Alcian blue staining. Briefly, the cells were fixed with 10% formaldehyde for at least 30 min, rinsed with distilled water followed by incubated in 0.0018 M H_2_SO_4_ for 30 min. Then, the acid solution was removed completely before adding Alcian blue solution (1% Alcian blue 8GX in 0.0018 M H_2_SO_4_). The staining step took 3 h, followed immediately by washing with 0.018 M H_2_SO_4_ for another 3 h to remove the redundant dye. Finally, the bound dye was eluted with dissociation buffer (4 M guanidine hydrochloride in 33% 1-propanol with 0.25% Triton X-100). The absorbance of each sample was then measured at 600 nm using a microplate reader in quadruplicate.

#### 4.2.6. Extracellular Matrix Formation of Cultured Meniscocytes

The cells of each group were fixed in 4% paraformaldehyde (Sigma-Aldrich, Inc., St. Louis, MO, USA) and dehydrated in a gradient ethanol series. Then, immunohistochemical staining was permeabilised in 0.1% (*v*/*v*) Triton X-100 (Sigma-Aldrich, Inc.), blocked with 5% (*v*/*v*) donkey serum (Biological), and rinsed in phosphate-buffered saline (PBS) (Gibco, Gaithersburg, MD, USA) containing 2% (*v*/*v*) bovine serum albumin BSA. Mouse monoclonal antibodies to collagen type I (Col I) and type II (Col II) (Calbiochem, Darmstadt, Germany) were utilized as primary antibodies in 1:1000 dilution followed by 3,3′-diaminobenzidine (DAB) secondary antibody (EnVision™, System-HRP, Dako, CA, USA) to assess the protein expression. The quantitative expression of protein deposition in each group was measured using Image J analytic software.

### 4.3. In Vivo

#### 4.3.1. Rabbit Meniscal Defect Repair

The protocol was approved by the Institutional Animal Care and Use Committee of National Taiwan University (IACUC approval number 99-092). Adult New Zealand White male rabbits (*n* = 18) were included and divided into three groups. The left knee in all rabbits was selected for study. All surgeries were performed using a standard surgical routine in the same operating room of the animal surgery centre by two senior surgeons (Chung-Hsun Chang and Tzong-Fu Kuo). Surgery was performed with an intramuscular (IM) injection of 50 mg/kg ketamine and 10 mg/kg xylazine anaesthesia. After shaving each animal’s bilateral knee joints, the surgical areas were then sterilized with iodine/alcohol. Under sterile conditions, the anterior horn and the body of the medial meniscus of the left knee of the hind legs was exposed through a complete arthrotomic incision. The patella was everted to allow maximum flexion of the joint. An oblique incision was made to create a 2.0 mm wedge shape full-thickness defect in the anterior portion of the medial meniscus. A small portion of meniscal tissue was removed leaving a defective site. The defects were divided into three groups according to treatment as follows: group A, defects were not sutured (non-suture group); group B, defects were sutured with 5–0 prolene (suture group); group C, defects were sutured with 5–0 prolene with PRF augmentation by implanting PRF fragments in or around the defect (PRF-augmented suture group), with six rabbits allocated to each group (Figure 6F–I). After surgery, the wound was irrigated, and the joint capsule, fascia, and skin were closed in layers. The animals were allowed to move within the cage without restriction after surgery.

#### 4.3.2. Semi-Quantitative Histological Score for Meniscal Repair Outcome

The rabbits were euthanized for histology, and the femurs were harvested and stored in 10% buffered formalin for histological analysis. Samples were then paraffin-embedded, sectioned with a microtome (5 μm), and stained with haematoxylin and eosin (HE). Later, three to five slides were chosen for each sample for histological scoring. An experienced pathologist blindly examined each slide under a light microscope. The pathologist was blinded to the treatment groups. All samples were semi-quantitatively graded using a four-stage meniscus score based on the HE-staining [49]. Major criteria listed in Table 1. The grading of degeneration was based on the pathologist’s decision regarding which bundle of criteria best fit most to the slides. The validity of the score was further examined by calculating the Cronbach’s alpha index which is 0.9157. 

### 4.4. Statistical Analysis

For the quantitative assay, each data point was derived from three independent experiments or an experiment of quadruplicate assay, and was presented as a mean with standard deviation. All analyses were performed using R 2.0.1 (R Project for Statistical Computing, available online: http://www.r-project.org/). Statistical significance was set at a *p* value of <0.05. The data were analysed using one-way ANOVA followed by a post hoc Scheffe test and a multiple comparisons Dunnett test. The statistical significances among the experimental groups are indicated by asterisks. Groups labelled with asterisk superscript letters indicate that the difference between the two groups is significant (*p* < 0.05).

## 5. Conclusions

In conclusion, the present study showed that PRF could promote meniscocytes migration, proliferation and matrix synthesis. The in vivo results suggest that PRF could facilitate the repair process of meniscal defects. Based on the results, a potential therapeutic approach to augment meniscal healing was proposed, which is expected to expedite the clinical translation of meniscal repair.

## Figures and Tables

**Table 1:** Hematoxylin-eosin staining based four-staged scoring system.

Grade	Criteria
0	Homogenous eosinophilic matrix staining No reduced cellularity Uniform morphology of chondrocytes (meniscocytes) No matrix clefts
1	Basophilic matrix staining Slight reduction of cellularity or small areas with reduced cellularity Small matrix clefts Minor polymorphism of chondrocytes (meniscocytes) Increased banding of fibrocartilage Possible accumulations of fat, minor chondroid metaplasia
2	Moderate basophilic matrix staining Obvious reduction of cellularity or multiple areas with reduced cellularity Matrix clefts Polymorphism of chondrocytes (meniscocytes) Banding of fibrocartilage Fat accumulations
3	Marked basophilic matrix staining Paucicellular, multiple obvious matrix clefts Marked polymorphism of chondrocytes (meniscocytes) and banding of fibrocartilage Fat accumulation
